# Understanding Electrochemical Alcohol Hydrogenolysis Enabled by Carbonyl Reduction in Lignocellulosic Biomass-Derived Aromatic Oxygenates

**DOI:** 10.1021/jacs.6c09300

**Published:** 2026-07-16

**Authors:** Myohwa Ko, Xin Yuan, Kwanpyung Lee, J. R. Schmidt, Kyoung-Shin Choi

**Affiliations:** Department of Chemistry, 5228 University of Wisconsin-Madison, Madison, Wisconsin 53706, United States

## Abstract

Molecules derived from lignocellulosic biomass are oxygenates with multiple oxygen-containing functional groups, such as hydroxyl and carbonyl groups. Therefore, the ability to selectively reduce a specific oxygenate group is essential for the reductive upgrading of such molecules. Previous studies on electrochemical biomass conversion have shown that alcohol hydrogenolysis, which involves cleavage of the σ­(C–O_alcohol_) bond, is extremely challenging for furfural and 5-hydroxymethylfurfural (HMF) derivatives, including furfuryl alcohol, 5-methylfurfuryl alcohol (MFA), and 2,5-bis­(hydroxymethyl)­furan (BHMF). In contrast, HMF itself undergoes alcohol hydrogenolysis relatively easily in acidic aqueous media. Considering that the only structural difference between HMF and BHMF or MFA is the presence of a carbonyl group, this observation raises the question of whether the carbonyl group in HMF facilitates alcohol hydrogenolysis. In this study, we designed systematic experiments to provide a coherent explanation of when and how a carbonyl group enables hydrogenolysis of a copresent alcohol group. Specifically, we show that alcohol hydrogenolysis in HMF proceeds via reduction of the carbonyl group to a ketyl radical, followed by a spin-center shift (SCS) through extended π conjugation. We also elucidate the effect of pH on the selectivity between carbonyl hydrogenation and alcohol hydrogenolysis, both of which share the ketyl radical intermediate. This mechanistic understanding enhances our ability to predict and control alcohol hydrogenolysis in the reductive upgrading of biomass-derived oxygenates.

## Graphical abstract

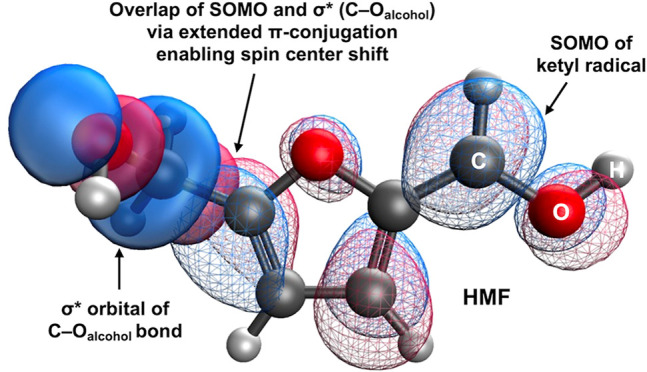

## Introduction

Lignocellulosic biomass has attracted considerable attention as a renewable, carbon-rich feedstock for the production of fuels and chemicals, offering a pathway to reduce the reliance on fossil resources.^1,2^ This interest stems from its natural abundance and its noncompetitive relationship with food supply chains.^3,4^ As a first step toward utilization, lignocellulosic biomass typically undergoes pyrolytic or hydrolytic depolymerization, yielding a wide range of oxygenated derivatives, including furanic and aryl compounds containing carbonyl and hydroxyl groups.^5–7^ The high oxygen content of these biomass-derived oxygenates reduces their energy density and limits their direct applicability as fuels; therefore, deoxygenation is essential for their conversion into fuels.^8,9^ Moreover, achieving deoxygenation selectively at specific sites among multiple oxygen-containing functional groups is crucial for selectively producing value-added chemicals from oxygenates.

Among various deoxygenation strategies, electrochemical reduction is particularly promising because it operates under ambient conditions and uses water as the hydrogen source, eliminating the need for preformed or in situ-generated H_2_.^8,10–13^ Moreover, it provides a platform for integrating renewable electricity into carbon-based molecular synthesis. Consequently, extensive research has focused on the electrochemical hydrogenation and hydrogenolysis of lignocellulosic biomass-derived molecules such as 5-hydroxymethylfurfural (HMF) and aromatic oxygenates.^14–20^

In previous efforts by our group and others to control the hydrogenation and hydrogenolysis of lignocellulosic biomass–derived oxygenates, notable differences were observed between alcohol and carbonyl hydrogenolysis. First, alcohol hydrogenolysis is considerably more difficult than aldehyde hydrogenolysis. For example, while the aldehyde group in furfural can undergo hydrogenolysis in acidic solutions, the alcohol group in furfuryl alcohol does not react under the same conditions (Figure 1a,b).^21–24^ Additionally, the alcohol groups in 2,5-bis­(hydroxymethyl)­furan (BHMF) and 5-methylfurfuryl alcohol (MFA) are inactive toward electrochemical alcohol hydrogenolysis (Figure 1c,d). In contrast, the alcohol group in HMF can undergo hydrogenolysis to form 5-methylfurfural (MF) and 2,5-dimethylfuran (DMF) in acidic solutions under reduction conditions where BHMF and MFA remain unreactive (Figure 1e).^19,25–29^ This is intriguing because the only structural difference between HMF and BHMF or MFA is the presence of a carbonyl group in HMF.

**Figure 1:**
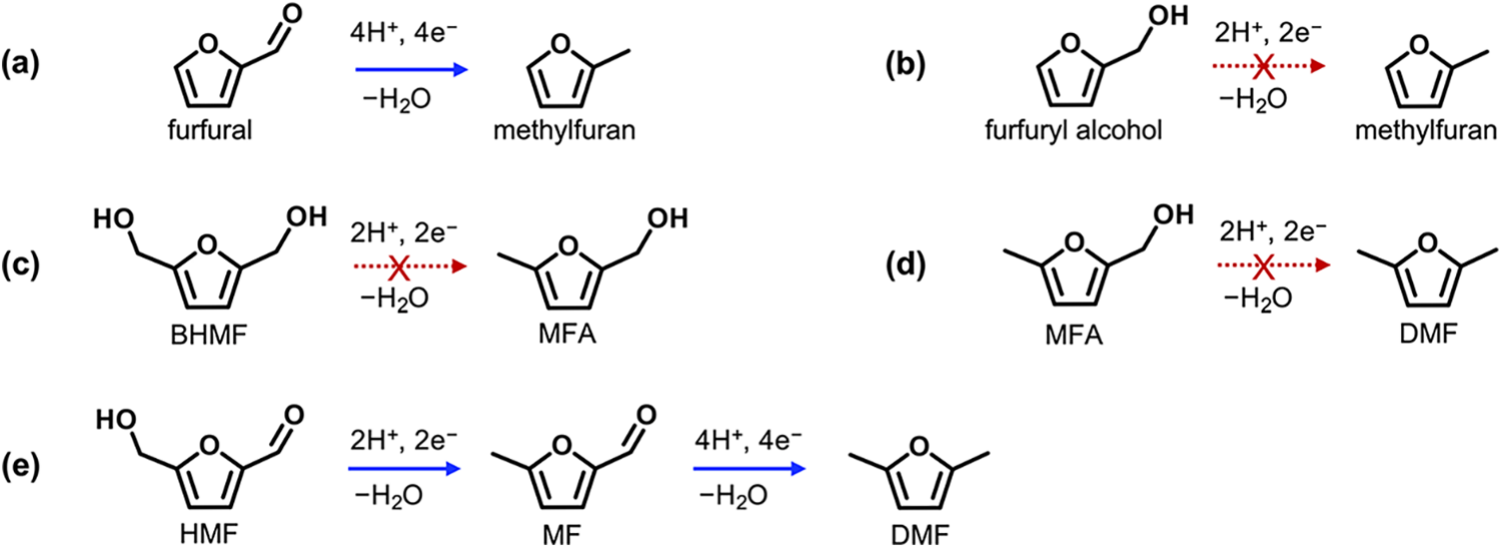
Feasibility of aldehyde or alcohol hydrogenolysis in (a) furfural, (b) furfuryl alcohol, (c) BHMF, (d) MFA, and (e) HMF.

These observations raise an important question: does the carbonyl group in HMF truly facilitate alcohol hydrogenolysis, and if so, which characteristics of the carbonyl group are responsible for this facilitation? In this study, we designed a series of systematic experiments to elucidate the role of the carbonyl group in promoting alcohol hydrogenolysis in HMF and related molecules. We then used our results to provide a coherent mechanistic understanding of when and how carbonyl reduction promotes or competes with alcohol hydrogenolysis. The insights gained from this study provide a foundation for predicting and controlling the electrochemical reactivity and selectivity in the reduction of carbonyl and alcohol groups copresent in lignocellulosic biomass–derived oxygenates and related organic molecules.

## Methods

### Materials

Cyclohexane (≥99.9%, Sigma-Aldrich), acetonitrile (≥99.9%, Sigma-Aldrich), toluene (99.8%, Sigma-Aldrich), 4-(hydroxymethyl)­benzaldehyde (4HMBAL) (98%, Ambeed), 1,4-benzenedimethanol (99%, Sigma-Aldrich), 4-methylbenzyl alcohol (98%, Sigma-Aldrich), *p*-tolualdehyde (97%, Sigma-Aldrich), *p*-xylene (99%, Sigma-Aldrich), (4-(trifluoromethyl)­phenyl)­methanol (98%, Ambeed), 4-(hydroxymethyl)­benzoic acid (98%, Ambeed), methyl 4-(hydroxymethyl)­benzoate (98%, Sigma-Aldrich), 3-(hydroxymethyl)­benzaldehyde (3HMBAL) (95%, Ambeed), 1,3-benzenedimethanol (98%, Ambeed), 3-methylbenzyl alcohol (98%, Oakwood), *m*-tolualdehyde (99%, Ambeed), *m*-xylene (99%, Oakwood), 2-hydroxyacetophenone (2HAP) (>98%, TCI America), acetophenone (≥98%, Sigma-Aldrich), 1-phenyl-1,2-ethanediol (98%, Ambeed), 1-phenylethanol (98%, Sigma-Aldrich), 2-phenylethanol (99%, Sigma-Aldrich), H_3_PO_4_ (≥85 wt %, Sigma-Aldrich), NaH_2_PO_4_ ·2H_2_O (≥99%, Sigma-Aldrich), Na_2_HPO_4_ (≥99%, Sigma-Aldrich), K_2_SO_4_ (99%, Thermo Scientific), H_2_SO_4_ (95–98%, Sigma-Aldrich), CuSO_4_·5H_2_O (≥98.0%, Sigma-Aldrich), and Cu foil (99.9%, Nimrod Copper Co.). All chemicals were used as received without further purification. Deionized water (Barnstead E-pure water purification system, resistivity >18 MΩ cm) was used for the preparation of all solutions.

### Electrode Preparation

A Cu foam electrode was used as the working electrode in this study because Cu is the most commonly used electrode for reduction studies of HMF and furfural derivatives.^30,31^ Both HER and dimerization of aromatic aldehydes are relatively insignificant on Cu compared to other metals,^31–34^ making it suitable for studying the electrochemical hydrogenation of aromatic carbonyl compounds. The high surface area of the foam electrode enhances the reduction rate, thereby reducing the time needed to produce reliable and quantifiable amounts of products.

The Cu foam electrode was prepared using a previously reported electrodeposition method (Figure S1).^35^ The electroplating bath consisted of an aqueous solution containing 0.16 M CuSO_4_ and 0.6 M H_2_SO_4_. A two-electrode setup, composed of Cu foil as both the working and counter electrodes, was used in an undivided cell. The Cu foam electrodes were electrodeposited galvanostatically at a current density of −2 A cm^–2^ for 5 s. The solution was magnetically stirred at 800 rpm throughout the deposition. After deposition, the electrode was rinsed with deionized water and dried under a N_2_ stream. Because the surface of Cu foam electrodes is susceptible to oxidation in air, the surface oxide layer was removed prior to each electrochemical experiment by immersing the electrode in a 10 wt % H_2_SO_4_ solution for approximately 10 s, followed by rinsing with deionized water and drying under a N_2_ stream. We also performed a postelectrolysis investigation of the Cu foam electrode (Figures S2–S3), and no obvious morphological changes were observed by scanning electron microscopy (Leo Supra55 VP) under the tested conditions.

### Electrolyte Preparation

Alcohol hydrogenolysis is promoted in acidic solutions. The reduction reactions of all reactants were investigated in a pH 1.4 phosphate buffer solution because, at this pH, two distinct reduction features were clearly resolved for all carbonyl compounds undergoing alcohol hydrogenolysis. Phosphate buffer solutions at pH 1.4 and pH 3.1 were prepared by mixing 0.5 M H_3_PO_4_ and 0.5 M NaH_2_PO_4_ solutions in the appropriate ratios until the desired pH was achieved. The pH 7 phosphate buffer solution was prepared by mixing 0.5 M NaH_2_PO_4_ and 0.5 M Na_2_HPO_4_ solutions in an appropriate ratio. The hydrogenation and hydrogenolysis of organic compounds consume protons, which can significantly increase the local pH during the reaction, particularly in mild pH solutions. Therefore, a considerable amount of buffer was used at all pH values to minimize pH drift during the reaction. The concentration of the organic reactant was 10 mM in all of the cases.

### Linear Sweep Voltammetry (LSV)

LSV was performed with a three-electrode setup in an H-type cell divided by a glass frit, without stirring, using a VSP multichannel potentiostat (BioLogic). The working electrode (WE) had an exposed surface area of 1 cm^2^. The counter electrode (CE) was Pt, and the reference electrode (RE) was a double-junction Ag/AgCl (4 M KCl) electrode, with saturated K_2_SO_4_ as the outer filling solution to prevent Cl^–^ contamination. The Pt CE was prepared by sputter-coating a 20 nm Ti adhesion layer followed by 100 nm of Pt onto a precleaned glass slide. The cathode compartment contained 10 mL of the electrolyte solution with the reactant, and the anode compartment contained the same volume of pure electrolyte solution. All LSVs were recorded by sweeping from the open-circuit potential in the negative direction at a scan rate of 50 mV s^–1^. Potentials recorded using the Ag/AgCl RE were converted to the reversible hydrogen electrode (RHE) scale using the following equation.

1 $$E_{\left(V\;\mathrm{vs}\;\mathrm{RHE}\right)}=E_{\left(V\;\mathrm{vs}\;\mathrm{Ag}/\mathrm{AgCl}\right)}+E_{\mathrm{Ag}/\mathrm{AgCl}\left(4\mathrm{M KCl}\right)}+\left(0.0591\;V\times \mathrm{pH}\right)$$

where *E*_Ag/AgCl(4M KCl)_ = 0.1976 V vs SHE at 25 °C

### Constant-Potential Electrolysis

Constant-potential electrolysis was carried out under the same conditions as the LSV measurements, but with magnetic stirring and a WE geometric surface area of 3.38 cm^2^. All electrolysis experiments were performed by passing the same amount of charge, equivalent to 2e^–^ per reactant molecule (19.3 C for 10 mL of a 10 mM solution). The conversion of each reactant can vary depending on the Faradaic efficiency (FE) for its reduction, which can impact the product distribution. However, the focus of our work is not to quantitatively analyze product distributions but to qualitatively assess the feasibility of hydrogenolysis of an alcohol group already present in the reactant. None of our results are ambiguous in this regard, as each reactant either produces a substantial or nondetectable amount of alcohol hydrogenolysis products. Thus, passing 2e^–^ per reactant molecule across all substrates is sufficient to achieve the goal of our study. For the electrolysis of 4HMBAL and 3HMBAL, a 5 mL cyclohexane extraction layer containing 1 μL of toluene as an internal standard was added to the cathode compartment to extract water-insoluble xylene.

The potential used for constant-potential electrolysis was selected independently for each reaction to minimize side reactions, ensuring that the majority of reactant consumption and charge transfer were directed toward carbonyl hydrogenation and alcohol hydrogenolysis, which are the focus of the current study. For example, −0.5 V vs RHE was chosen for the reduction of 4HMBAL and 3HMBAL because radical dimerization (e.g., ketyl radical dimerization to form pinacol products) is considerable for aromatic aldehydes at less negative potentials (Figure S4a,b).^34^ Because dimerization is not completely suppressed at −0.5 V vs RHE, it accounts for cases in which the absolute selectivities of the carbonyl hydrogenation and alcohol hydrogenolysis products do not sum to 100%. In contrast, 2HAP does not undergo radical dimerization (Figure S4c); therefore, a less negative potential (−0.3 V vs RHE) was selected to minimize the hydrogen evolution reaction (HER), which increases at more negative potentials.

### Product Analysis

Quantification of soluble products was performed using high-performance liquid chromatography (HPLC, Prominence-i LC 2030C 3D, Shimadzu) equipped with a Restek Roc C18 column (150 mm × 4.6 mm, 5 μm particle size) and a mobile phase composed of solvent A (water, 0.1% phosphoric acid) and solvent B (acetonitrile, 0.1% phosphoric acid). The column temperature was maintained at 40 °C, with a mobile phase flow rate of 1 mL min^–1^. For 4HMBAL and 3HMBAL analysis, gradient separation began with 10% solvent B for 4 min, which was increased to 80% over 8 min, held at this composition for 2 min, and then returned to the initial composition. For 2HAP analysis, gradient separation was initiated with 20% solvent B. Quantification was achieved by comparing the integration of PDA absorbances at multiple wavelengths to the calibration curves of the corresponding products. After the required charge had passed, aliquots were taken from the cathode compartment for HPLC analysis without further treatment.

To detect water-insoluble products (xylene), the cyclohexane extraction layer was analyzed using gas chromatography–mass spectrometry (GC-MS, QP2010, Shimadzu) equipped with a ZB-5 ms capillary column (30 m × 0.25 mm i.d., 0.25 μm film thickness). An injection volume of 1 μL was used with a split ratio of 20:1, and the injector temperature was maintained at 250 °C. High-purity helium (99.999%) served as the carrier gas at a constant flow rate of 1.50 mL min^–1^. The column oven temperature was initially set at 40 °C for 3.55 min, increased at 20 °C min^–1^ to 230 °C, and then held at 230 °C for 1.25 min. Chromatogram data were collected after the elution of the cyclohexane solvent peak. Product quantification was performed using the internal standard method, with calibration curves generated from a series of standard solutions of known concentration. In addition to xylene, a portion of methylbenzyl alcohol and the majority of tolualdehyde partitioned into the cyclohexane layer. Therefore, the total product amount was determined by combining the quantities obtained from HPLC and GC analyses. The identity of each GC peak was further confirmed by mass fragmentation patterns that matched those of commercial standards. The HPLC and GC chromatograms of the reactants and reduction products are shown in the SI (Figures S5–S8).

Absolute selectivity (%) and FE (%) were calculated using the following equations.

2 $$a\mathrm{bsolute selectivity}\;\left(\%\right)=\frac{\mathrm{mol of specific product}}{\mathrm{mol of consumed reactant}}\times 100$$

3 $$\mathrm{FE}\;\left(\%\right)=\frac{\mathrm{mol}\;\mathrm{of}\;e^{-}\;\mathrm{consumed}\;\mathrm{to}\;\mathrm{produce}\;\mathrm{specific}\;\mathrm{product}}{\mathrm{mol}\;\mathrm{of}\;e^{-}\;\mathrm{passed}}\times 100$$

The FEs of the reduction products discussed in the main text are summarized in Tables S1–S3.

### Natural Bond Orbital (NBO) Analysis

In order to investigate the role of the ketyl radical in alcohol hydrogenolysis through orbital overlap, NBO analysis was performed using NBO 7.0 ^36^ implemented in Gaussian 16 ^37^ to construct the σ*­(C–O_alcohol_) orbital, while the singly occupied molecular orbital (SOMO) of the ketyl radical was obtained directly from Gaussian 16. The Perdew–Burke–Ernzerhof (PBE) functional^38^ with the def2-TZVP basis set^39^ was used.

## Results and Discussion

### Reduction of 4HMBAL

We began our investigation with 4-(hydroxymethyl)­benzaldehyde (4HMBAL) to assess whether the reduction behaviors of alcohol and carbonyl groups observed in HMF and its derivatives can be generalized to other molecules. We selected 4HMBAL as the model compound because it contains both a hydroxymethyl group and a carbonyl group attached to an aromatic ring, similar to HMF. Furthermore, its derivatives—either lacking one of these groups or containing identical or different functional groups at various positions—are commercially available, allowing systematic control experiments to examine how structural variations in 4HMBAL influence its electrochemical reactivity.

The molecular structures of 4HMBAL and its derivatives used in the first set of experiments are shown in Figure 2a. We first recorded the LSVs of these molecules using a Cu foam electrode (Figure S1) in a phosphate-buffered solution (pH 1.4).

**Figure 2:**
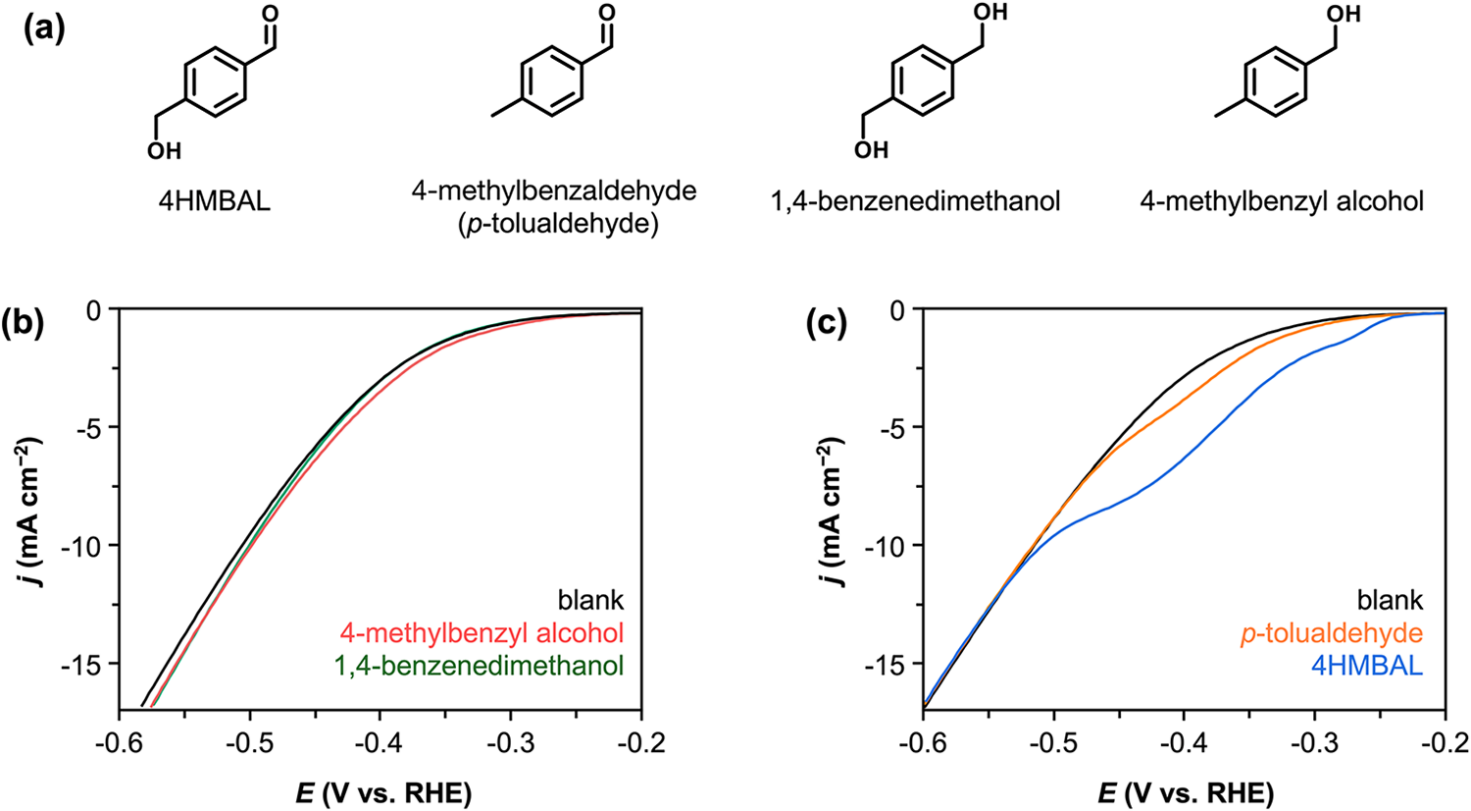
(a) Molecular structures of 4HMBAL and related compounds used for comparison. (b) LSVs recorded in the absence of reactant (blank) and in the presence of 10 mM 4-methylbenzyl alcohol or 1,4-benzenedimethanol. (c) LSVs recorded in the absence of reactant (blank) and in the presence of 10 mM *p*-tolualdehyde or 4HMBAL. All measurements were performed using a Cu foam electrode in a pH 1.4 buffer solution.

In the absence of any reactants (blank solution), only the hydrogen evolution reaction (HER) current was observed, starting at approximately −0.25 V vs reversible hydrogen electrode (RHE). When 10 mM 4-methylbenzyl alcohol (containing one hydroxyl group) or 10 mM 1,4-benzenedimethanol (containing two hydroxyl groups) was present, only the HER reduction feature was observed, indicating that the alcohol groups in these compounds do not undergo electrochemical reduction prior to HER (Figure 2b). Furthermore, constant-potential reduction of these molecules at −0.5 V vs RHE, to test whether they could be reduced after the onset of HER, resulted in no detectable products (Table S4). These results confirm that these molecules behave similarly to MFA and BHMF.

In contrast, the LSV of *p*-tolualdehyde, which contains a carbonyl group, shows a cathodic wave before the HER onset, corresponding to carbonyl reduction, indicating that carbonyl reduction in *p*-tolualdehyde occurs more readily than the HER (Figure 2c). The LSV of 4HMBAL, which contains both a carbonyl and an alcohol group, shows two cathodic waves before the HER (Figure 2c), suggesting that both functional groups may undergo reduction prior to the HER. Since Figure 2b shows that the alcohol group alone does not exhibit any reduction feature, it is reasonable to presume that alcohol hydrogenolysis occurs only in the presence of the carbonyl group. Accordingly, the first reduction wave (appearing at a less negative potential) should be at least partly associated with alcohol hydrogenolysis of 4HMBAL in the presence of the carbonyl group. The second wave, between the first wave and the HER, corresponds to carbonyl reduction of *p*-tolualdehyde generated in situ via alcohol hydrogenolysis of 4HMBAL during the first peak.

When constant-potential electrolysis of 4HMBAL was performed at −0.5 V vs RHE, multiple reduction products were detected (Figure 3a), including *p*-tolualdehyde and *p*-xylene, which cannot form without alcohol hydrogenolysis of 4HMBAL (Figure 3a). This result confirms that the carbonyl group in 4HMBAL facilitates alcohol hydrogenolysis, similar to HMF. A considerable amount of 4-methylbenzyl alcohol was also observed; however, this product can result from both carbonyl hydrogenolysis of 4HMBAL and carbonyl hydrogenation of *p*-tolualdehyde formed via alcohol hydrogenolysis (Figure 3b). Therefore, 4-methylbenzyl alcohol cannot serve as direct evidence of alcohol hydrogenolysis. A small amount of 1,4-benzenedimethanol was also detected, indicating that carbonyl hydrogenation of 4HMBAL prior to alcohol hydrogenolysis occurs to a minor extent.

**Figure 3:**
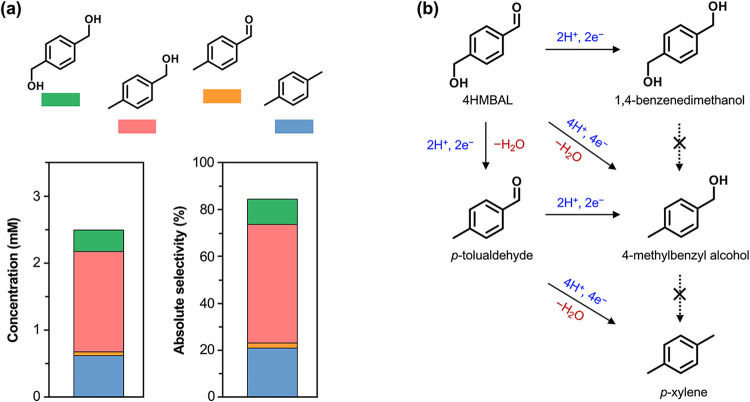
(a) Quantification of products obtained from constant-potential electrolysis of 10 mM 4HMBAL in a pH 1.4 buffer solution at −0.5 V vs RHE using a Cu foam electrode after passing the amount of charge equivalent to 2e^–^ per 4HMBAL molecule. (b) Multiple electrochemical reduction pathways of 4HMBAL.

### Replacing the Aldehyde Group with Other EWGs

The observation that the presence of a carbonyl group promotes alcohol hydrogenolysis–otherwise electrochemically inactive–in HMF and 4HMBAL prompted us to consider which characteristics of the carbonyl group facilitate this reaction. We first hypothesized that it might be the electron-withdrawing nature of the carbonyl group, which can pull electron density from the carbon in the C–O_alcohol_ bond, increasing its electrophilicity and thereby activating the bond toward reduction.

To test this hypothesis, we replaced the aldehyde group of 4HMBAL with other electron-withdrawing groups (EWGs),^40^ such as −CF_3_, −COOH, and −COOCH_3_ (Figure 4a). However, LSVs of the resulting molecules showed no additional reduction features before HER (Figure 4b–d). Constant-potential electrolysis of these molecules at −0.5 V vs RHE also resulted in negligible changes in the concentrations of the starting compounds and no products from alcohol hydrogenolysis (Table S5). Because −CF_3_, −COOH, and −COOCH_3_ are all more electron-withdrawing than the aldehyde group, these results suggest that the electron-withdrawing ability of the aldehyde group is not responsible for promoting alcohol hydrogenolysis in HMF and 4HMBAL. Furthermore, because −COOH and −COOCH_3_ also contain a carbonyl group, our results indicate that attaching an −OH or −OCH_3_ group to the carbonyl carbon diminishes or abolishes the carbonyl group’s ability to enable alcohol hydrogenolysis.

**Figure 4:**
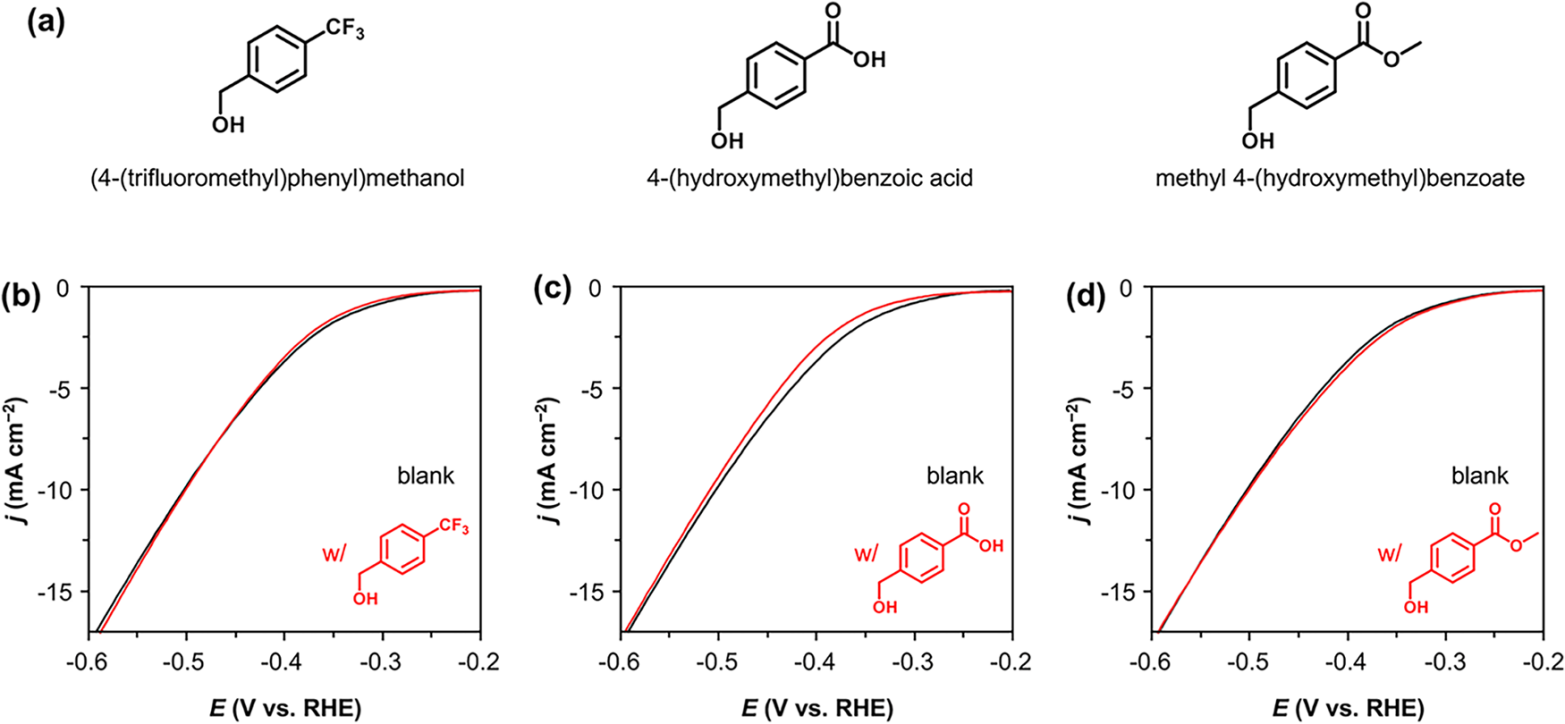
(a) Molecules obtained by replacing the aldehyde group with other EWGs. LSVs recorded in the presence of 10 mM (b) (4-(trifluoromethyl)­phenyl)­methanol, (c) 4-(hydroxymethyl)­benzoic acid, and (d) methyl 4-(hydroxymethyl)­benzoate, as well as in the absence of any reactant (blank). All measurements were performed using a Cu foam electrode in a pH 1.4 buffer solution.

### Changing the Carbonyl Group Location

Next, we investigated whether the position of the carbonyl group relative to the alcohol group affects reactivity by testing 3-(hydroxymethyl)­benzaldehyde (3HMBAL), in which the CH_2_OH group is attached at the *meta* position rather than the *para* position of the benzaldehyde. Unlike 4HMBAL, the LSV of 3HMBAL exhibited only a single reduction wave before the HER onset (Figure 5a). The position and shape of this reduction wave closely match those of *m*-tolualdehyde, which lacks a hydroxyl group, suggesting that the hydroxyl group in 3HMBAL is likely electrochemically inactive and that the observed reduction feature corresponds solely to the carbonyl reduction.

**Figure 5:**
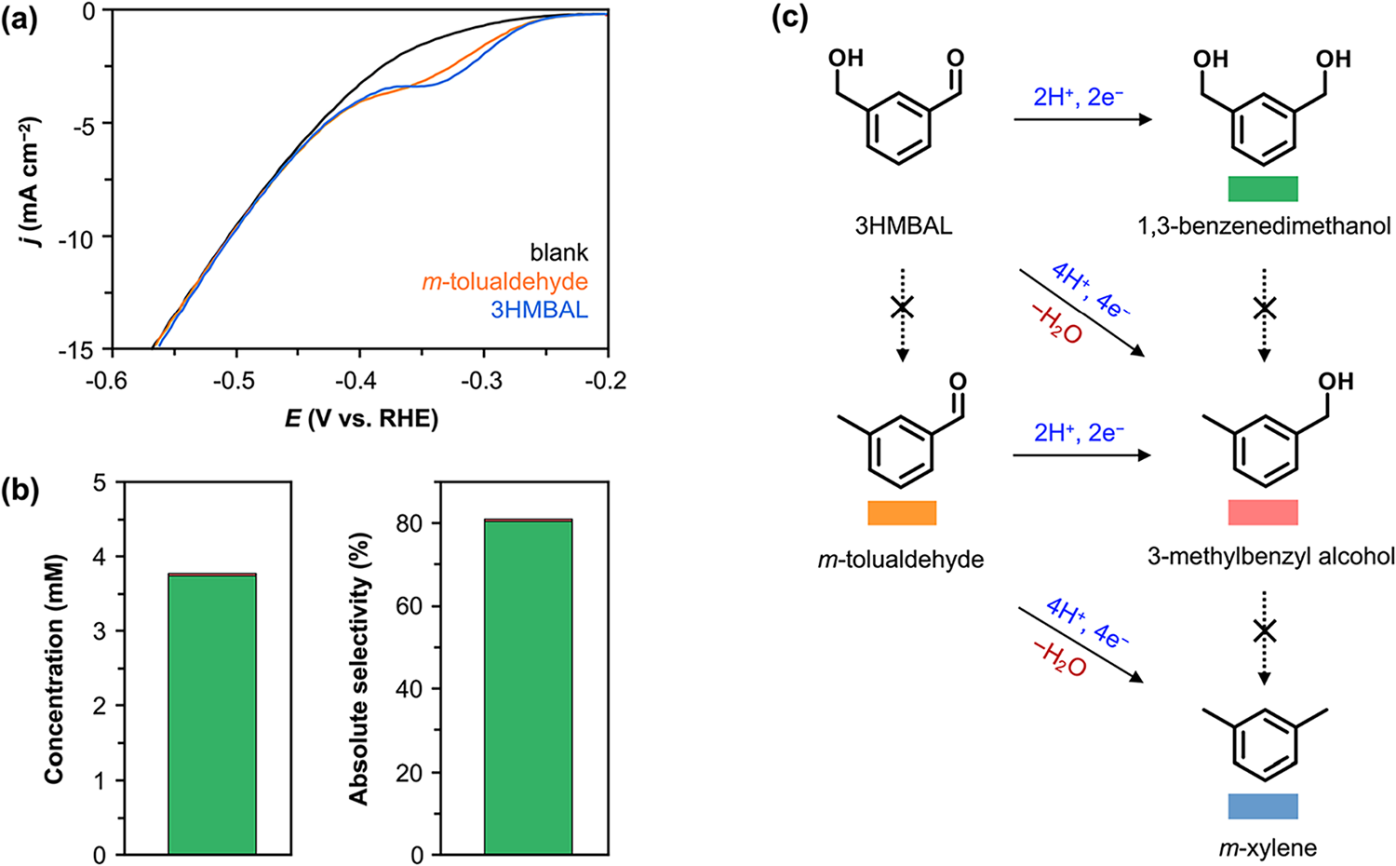
(a) LSVs recorded in the presence of 10 mM *m*-tolualdehyde or 3HMBAL, as well as in the absence of any reactant (blank). (b) Quantification of products obtained from constant-potential electrolysis of 10 mM 3HMBAL at −0.5 V vs RHE, after passing the amount of charge equivalent to 2e^–^ per 3HMBAL molecule. (c) Proposed electrochemical reduction pathways of 3HMBAL. All measurements were performed using a Cu foam electrode in a pH 1.4 buffer solution.

Constant-potential electrolysis of 3HMBAL at −0.5 V vs RHE primarily generated the carbonyl hydrogenation product, 1,3-benzenedimethanol (Figure 5b). Neither *m*-tolualdehyde nor *m*-xylene, which would require alcohol hydrogenolysis (Figure 5c), was detected. A small amount of 3-methylbenzyl alcohol was also observed. While 3-methylbenzyl alcohol could, in principle, be formed either with or without alcohol hydrogenolysis, we found no indication of *m*-tolualdehyde formation. We therefore infer that, in this case, 3-methylbenzyl alcohol was most likely produced via carbonyl hydrogenolysis of 3HMBAL rather than through alcohol hydrogenolysis.

The results obtained from 4HMBAL and 3HMBAL indicate that the promotion of alcohol hydrogenolysis in the presence of a carbonyl group requires a specific spatial arrangement between the hydroxyl and carbonyl groups. This suggests that the electronic conjugation between these groups plays a key role. On the basis of this insight, we developed a mechanistic model in which alcohol hydrogenolysis occurs not through direct reduction of the hydroxyl group but via 1e^–^ reduction of the carbonyl group followed by a spin-center shift (SCS), as illustrated in Figure 6. SCS, defined as a 1,2-radical shift accompanied by elimination of an adjacent leaving group, is a well-established concept in biological and synthetic organic chemistry,^41^ although it has never been employed to interpret results obtained from the electrochemical reduction of biomass-derived oxygenates and their structural analogues.

**Figure 6:**
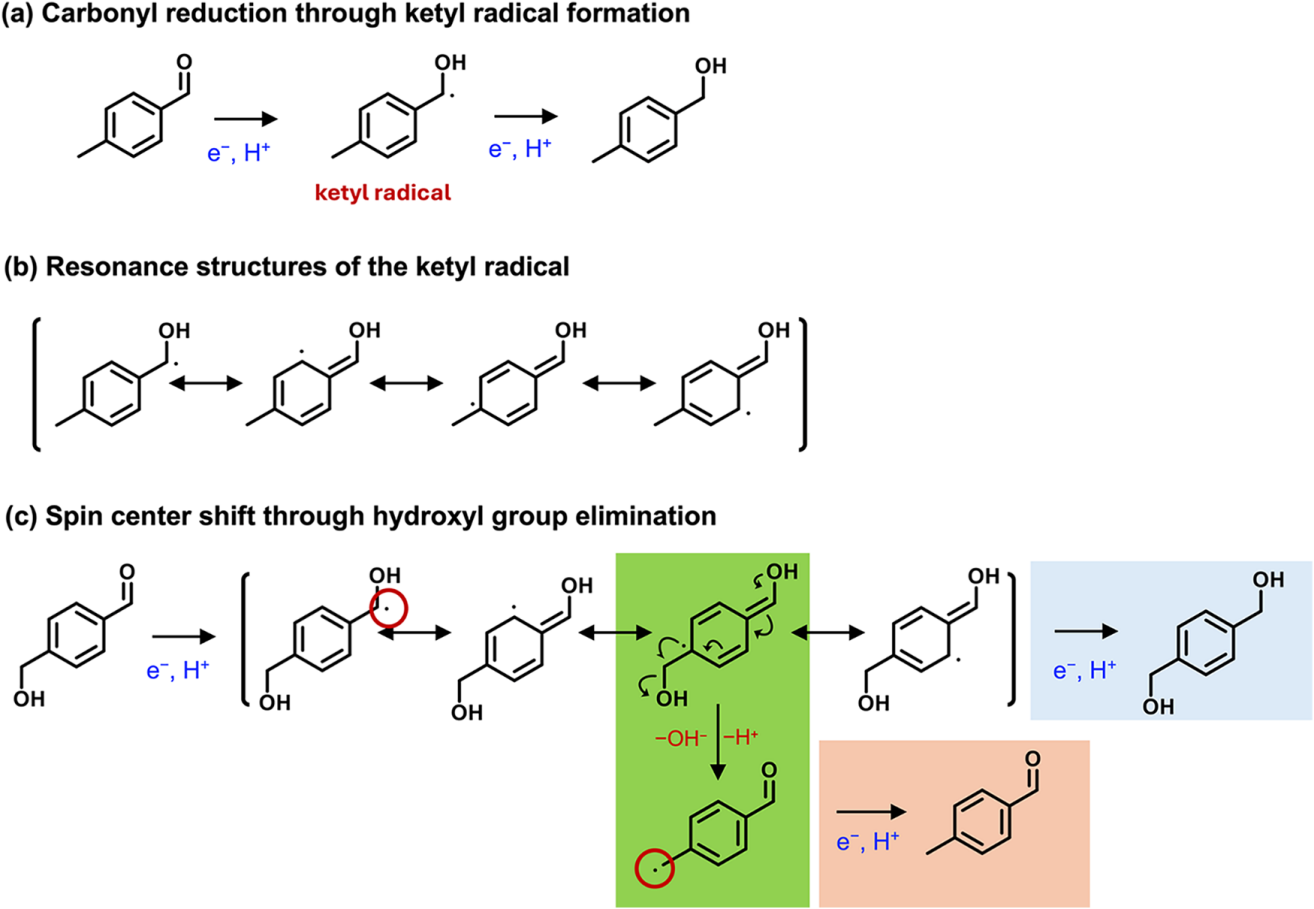
(a) Two-step reduction pathway for carbonyl hydrogenation via ketyl radical formation, (b) resonance structures of the ketyl radical across the aromatic ring, and (c) spin-center shift (SCS) from the ketyl radical to the −CH_2_• radical at the *para* position through elimination of the hydroxyl group in −CH_2_OH (green box). The resulting −CH_2_• radical can be further reduced to −CH_3_ (orange box), whereas the ketyl radical that does not undergo SCS can be further reduced to yield a carbonyl hydrogenation product (blue box). For simplicity, the electrode surface that stabilizes the reactants and intermediates is not shown.

It is well established from previous studies that the reduction of a carbonyl group begins with the formation of a ketyl radical (Figure 6a).^17,27^ Notably, the p*K*_a_ of the neutral ketyl radical formed from benzaldehyde has been reported as 8.4.^42^ Assuming a similar p*K*_a_ for ketyl radicals derived from other aromatic aldehydes, the ketyl radical anion formed by one-electron reduction of the carbonyl group must be protonated in acidic solutions to form a neutral ketyl radical. Accordingly, we indicate in Figure 6a that the first reduction step of a carbonyl group to form a ketyl radical requires both electron and proton transfers. When the ketyl radical is attached to the aromatic ring, it can adopt resonance structures, as shown in Figure 6b, locating a resonantly equivalent carbon radical at the *para* and *ortho* positions of the benzene ring. (The radicals at different locations in Figure 6b are not considered SCS, as they represent resonantly equivalent radicals.)

When a −CH_2_OH group is attached at the *para* position, the singly occupied molecular orbital (SOMO) of the carbon radical at the *para* position (essentially the ketyl radical) overlaps with the σ* orbital of the adjacent C–O_alcohol_ bond, thereby weakening this bond, which is supported by our natural bond orbital (NBO) analysis (Figure S9a). This interaction facilitates the elimination of the hydroxyl group and shifts the spin center (the radical) from C_para_ (essentially the ketyl radical) to the methyl carbon, forming −CH_2_• (green box in Figure 6c, with radicals involved in SCS indicated by red circles). Notably, during this elimination of the hydroxyl group, electron donation from O_carbonyl_ is critical to prevent the formation of an unstable carbocation at the methyl carbon. This electron shift from O_carbonyl_ leads to deprotonation of the protonated ketyl radical and consequently regenerates the carbonyl group.

Overall, the SCS process shown in Figure 6c involves removal of OH^–^ from the −CH_2_OH group and H^+^ from the protonated ketyl radical. Such SCS occurs because it lowers the system’s free energy, meaning it is a spontaneous reaction.^41^ We expect that the eliminated hydroxyl group would be rapidly protonated by the acidic electrolyte to form H_2_O. In Figure 6c, we represented the removal of a hydroxyl group from the methyl carbon and the removal of H^+^ from the ketyl radical separately, rather than as the elimination of H_2_O, to indicate that they are coupled but spatially separated events.

The methyl carbon radical formed via SCS and the ketyl radical that does not undergo SCS can each undergo a second reduction step, producing the alcohol hydrogenolysis and carbonyl hydrogenation products, respectively (orange and blue boxes in Figure 6c).

We note that the resonance structures of the ketyl radical (Figure 6b) do not include a carbon radical at the *meta* position. As a result, the overlap between the SOMO of the ketyl radical and the σ* orbital of the adjacent C–O_alcohol_ bond–crucial for weakening this bond–cannot occur through π-conjugation in 3HMBAL, as confirmed by our NBO analysis (Figure S9b). This explains why the hydroxyl group in the −CH_2_OH substituent at the *meta* position in 3HMBAL does not undergo alcohol hydrogenolysis, unlike the *para*-substituted −CH_2_OH group in 4HMBAL (Figure 5).

### SCS Not through the Aromatic Ring

Figure 6b shows that the ketyl radical and the carbon radical at the *para* position in the benzene ring are equivalent through resonance. The carbon radical at the *para* position (essentially the ketyl radical) can shift to the carbon in the −CH_2_OH group attached at that position via elimination of the hydroxyl group. This indicates that reductive elimination of a hydroxyl group via SCS should also be possible when a −CH_2_OH group is directly attached to the carbonyl carbon.

To test this hypothesis, we examined 2-hydroxyacetophenone (2HAP), in which a hydroxyl group is attached to the α-carbon of the carbonyl group (equivalent to a −CH_2_OH group attached to the carbonyl carbon). Our proposed model illustrating how the ketyl radical in 2HAP can facilitate alcohol hydrogenolysis via SCS is shown in Figure 7a. This mechanism is analogous to that in Figure 6c but does not involve extended π-conjugation between the carbonyl group and the carbon bearing the hydroxyl group. Our computational result showing the overlap between the SOMO of the ketyl radical and σ*­(C–O_alcohol_) orbital is presented in Figure S9c.

**Figure 7:**
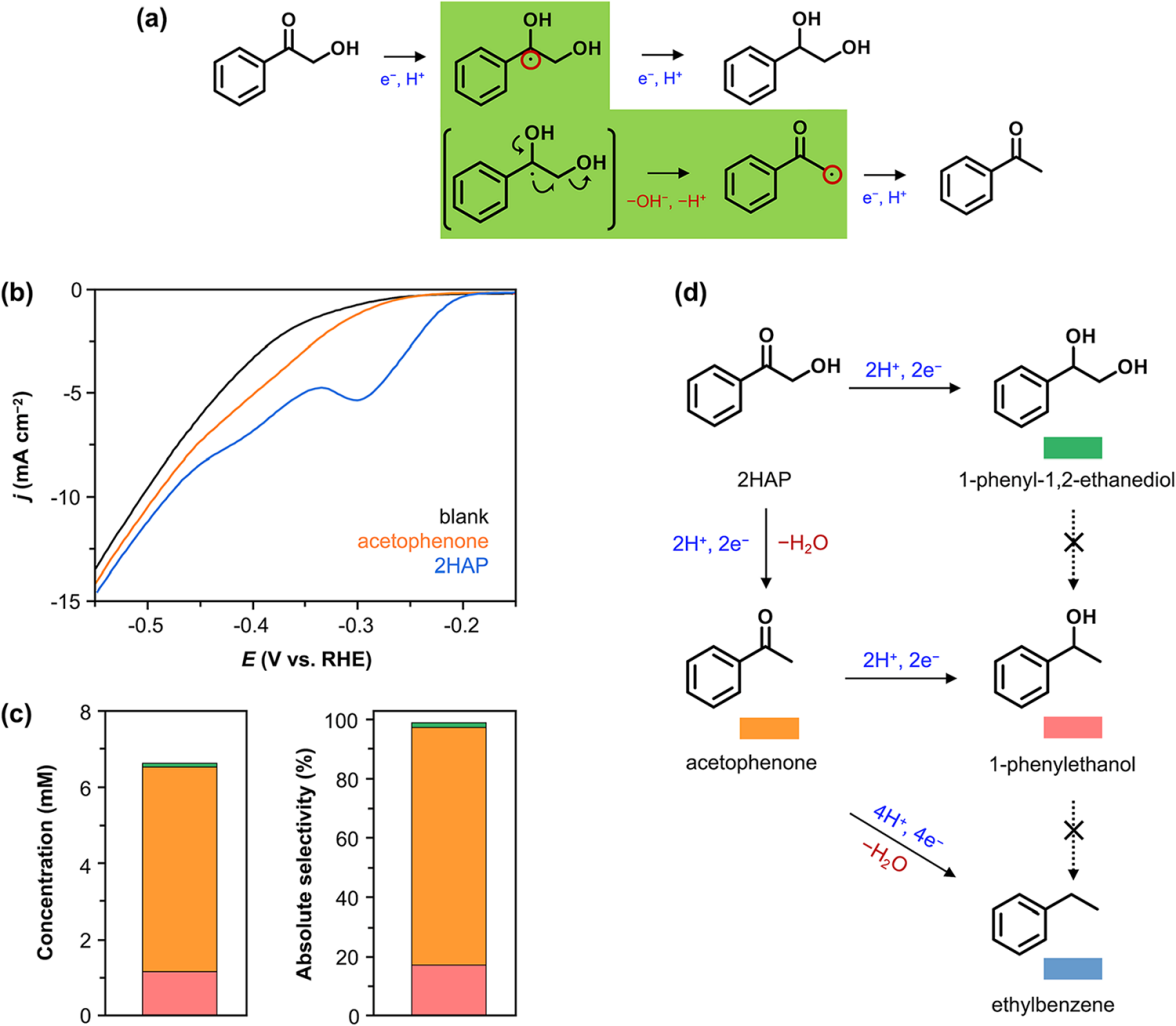
(a) Proposed model illustrating how the ketyl radical formed from 2HAP can lead to both carbonyl hydrogenation (top) and alcohol hydrogenolysis (bottom). (b) LSVs recorded in the presence of 10 mM acetophenone or 2HAP, as well as in the absence of reactant (blank). (c) Quantification of products obtained from constant-potential electrolysis of 10 mM 2HAP at −0.3 V vs RHE after passing the amount of charge equivalent to 2e^–^ per 2HAP molecule. (d) Electrochemical reduction pathways of 2HAP. All measurements were conducted using a Cu foam electrode in a pH 1.4 buffer solution.

The LSVs of 2HAP and acetophenone, which is structurally identical to 2HAP but lacks a hydroxyl group, are overlaid in Figure 7b. While acetophenone shows only one reduction wave corresponding to carbonyl reduction prior to the onset of the HER, 2HAP exhibits two reduction waves. The first feature should be at least partly associated with the elimination of the hydroxyl group in 2HAP, and the second feature should be associated with the reduction of the carbonyl group of acetophenone, which is generated in situ via the alcohol hydrogenolysis of 2HAP during the first reduction wave. These LSV behaviors closely resemble those of 4HMBAL and *p*-tolualdehyde shown in Figure 2c.

Constant-potential reduction of 2HAP at −0.3 V vs RHE yielded acetophenone as the major product (Figure 7c,d), and a portion of the acetophenone was further reduced to 1-phenylethanol via carbonyl reduction. This observation–that a hydroxyl group directly attached to the C_α_ of a carbonyl group can undergo reductive elimination–demonstrates that extended π-conjugation between the carbonyl and hydroxyl groups is not essential for alcohol hydrogenolysis via SCS. The sole role of the extended π-conjugation in 4HMBAL is to render the OH group in the para-substituted CH_2_OH moiety resonantly equivalent to the OH group attached to the C_α_ of the carbonyl group, thereby enabling SCS. Thus, the essential requirement for enabling alcohol hydrogenolysis via ketyl radical formation followed by SCS is a spatial arrangement of the carbonyl and alcohol groups equivalent to that in an α-ketol, where the hydroxyl group is attached to the C_α_ of the carbonyl group. We also detected a small amount of 1-phenyl-1,2-ethanediol, the product formed when carbonyl hydrogenation occurs prior to alcohol hydrogenolysis, as in the case of 4HMBAL.

We additionally confirmed that 2-phenylethanol and 1-phenyl-1,2-ethanediol, structural analogues of 2HAP lacking a carbonyl group, exhibit no additional reduction waves prior to the HER onset in their LSVs (Figure S10) and show no conversion during constant-potential electrolysis at −0.5 V vs RHE (Table S6). These results further confirm that the reductive elimination of the hydroxyl group in 2HAP occurs through reduction of the carbonyl group.

### Selectivity between Carbonyl Hydrogenation and Alcohol Hydrogenolysis

The understanding that the ketyl radical serves as the common intermediate for both carbonyl hydrogenation and alcohol hydrogenolysis of α-ketols and their structural equivalents enables us to identify a key factor that affects the selectivity between these two processes, as illustrated in Figure 8a.

**Figure 8:**
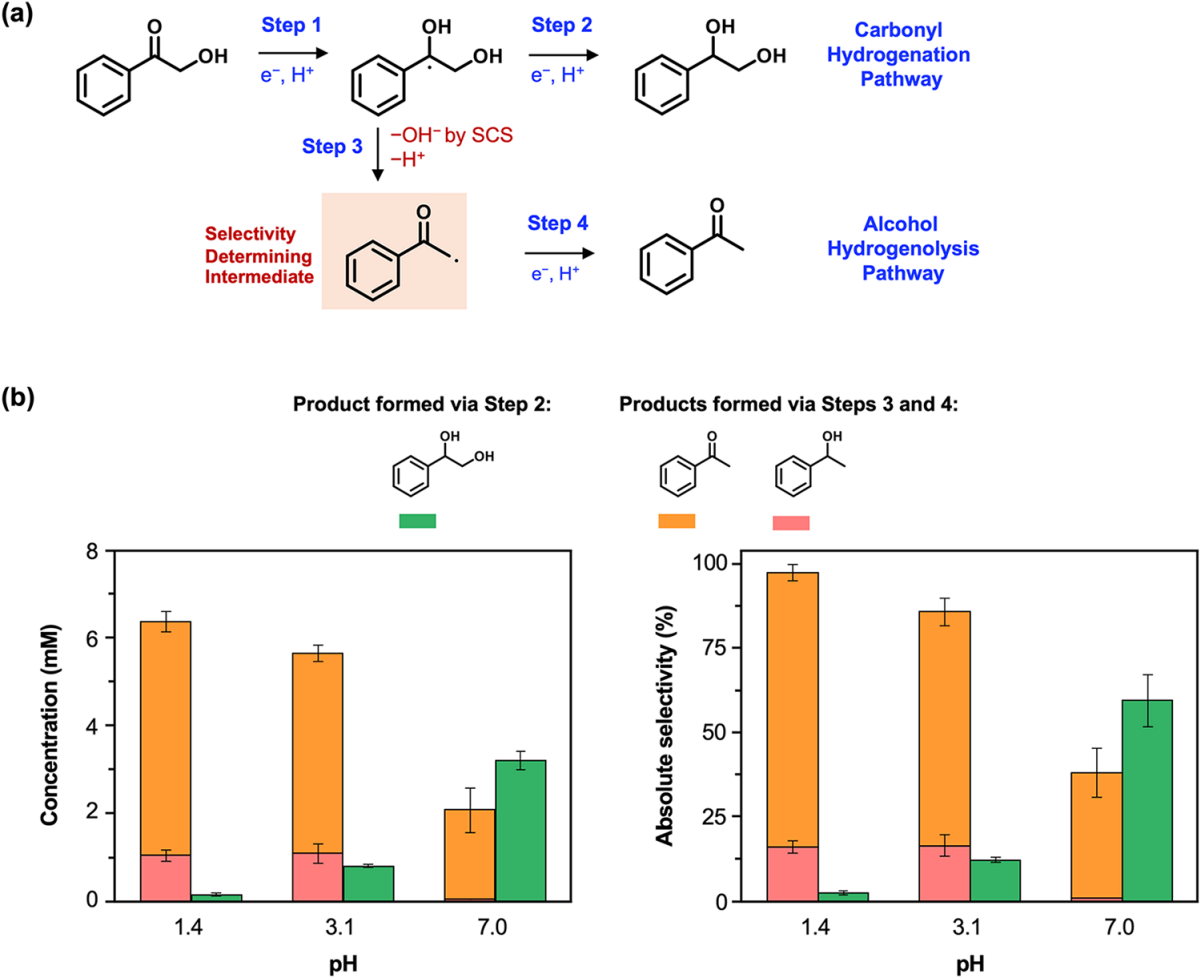
(a) Illustration of the selectivity-determining step during 2HAP reduction. (b) Quantification of products obtained from constant-potential electrolysis of 10 mM 2HAP at −0.3 V vs RHE after passing the amount of charge equivalent to 2e^–^ per 2HAP molecule using a Cu foam electrode in pH 1.4, 3.1, and 7.0 buffer solutions.

After the ketyl radical is formed in the first reduction step of the carbonyl group (Step 1), it can undergo a second reduction step to yield an alcohol–the product of carbonyl hydrogenation (Step 2). However, if SCS occurs via hydroxyl elimination (Step 3) before Step 2, the ketyl radical is converted into a C_α_ radical, thereby interrupting the carbonyl hydrogenation pathway. When the C_α_ radical subsequently undergoes the second reduction step (Step 4), the alcohol hydrogenolysis product is obtained. Thus, the feasibility of Step 3 is a critical factor influencing the selectivity between carbonyl hydrogenation and alcohol hydrogenolysis. When Step 3 is highly feasible, high selectivity toward alcohol hydrogenolysis is expected; conversely, if Step 3 is not feasible, carbonyl hydrogenation becomes the dominant pathway.

To confirm the validity of the proposed model in Figure 8a, we investigated how changing the pH affects the feasibility of Step 3 and its impact on the selectivity between carbonyl hydrogenation and alcohol hydrogenolysis. Our results with 2HAP and 4HMBAL in acidic solutions showed that the carbonyl hydrogenation product formed only as a minor product, indicating that Step 3 was facile in both cases. However, we note that the hydroxyl group is not a good leaving group, and Step 3 in these solutions was likely facilitated by the abundant protons near the hydroxyl group acting as effective acceptors. On the basis of this understanding, we predicted that the feasibility of Step 3 would decrease as pH increases, leading to lower selectivity for alcohol hydrogenolysis.

To verify this postulation, we examined whether the selectivity of alcohol hydrogenolysis of 2HAP changes with pH. The products obtained from 2HAP reduction at pH 1.4, 3.1, and 7.0 are compared in Figure 8b. The results show that as pH increases, the selectivity for products that underwent Steps 3–4 decreases, while the selectivity for the product that underwent Step 2 correspondingly increases. We repeated the same experiment with 4HMBAL and observed the same trend (Figure S11). These findings confirm that the feasibility of Step 3 is influenced by pH, making pH a critical factor in determining alcohol hydrogenolysis selectivity in α-ketols and their equivalents.

Our understanding that the ketyl radical serves as the common intermediate for both carbonyl hydrogenation and alcohol hydrogenolysis in α-ketols and their equivalents also provides new insights into the interpretation of the LSVs of 4HMBAL and 2HAP presented above. The two reduction features observed in the LSVs of 4HMBAL and 2HAP are not due to the presence of two independent reduction centers–the alcohol and carbonyl groups–in these molecules. Instead, both reduction features arise from carbonyl reduction: the first corresponds to the carbonyl reduction of the original reactants (4HMBAL and 2HAP), and the second corresponds to the carbonyl reduction of the in situ–generated alcohol hydrogenolysis products (*p*-tolualdehyde and acetophenone). The latter are reduced at more negative potentials because the –CH_3_ group is more electron-donating than the –CH_2_OH group, making the carbonyl group less electrophilic (Figures 2c and 7b). Thus, when selectivity for alcohol hydrogenolysis is appreciable during the first reduction wave, carbonyl reduction of the resulting alcohol hydrogenolysis product emerges as the second reduction feature.

### Alcohol Hydrogenolysis in HMF and BHMF

Combining the results and understanding presented above, we now explain why alcohol hydrogenolysis is feasible in HMF but not in BHMF.^25–27,43^ Our proposed mechanistic model for alcohol hydrogenolysis in HMF is illustrated in Figure 9a. This model shows that the C5 position of the furan ring, where the −CH_2_OH group is attached, is resonantly equivalent to the carbonyl carbon when the ketyl radical is formed. Thus, upon ketyl radical generation, its SOMO can overlap with the σ* orbital of the C–O_alcohol_ bond through extended π-conjugation (Figure S9d), thereby weakening the C–O_alcohol_ bond. In other words, the spatial arrangement of the carbonyl and hydroxyl groups in HMF is equivalent to that of an α-ketol, as in 4HMBAL. As a result, ketyl radical formation in HMF could be followed by SCS, enabling hydroxyl group elimination.

**Figure 9:**
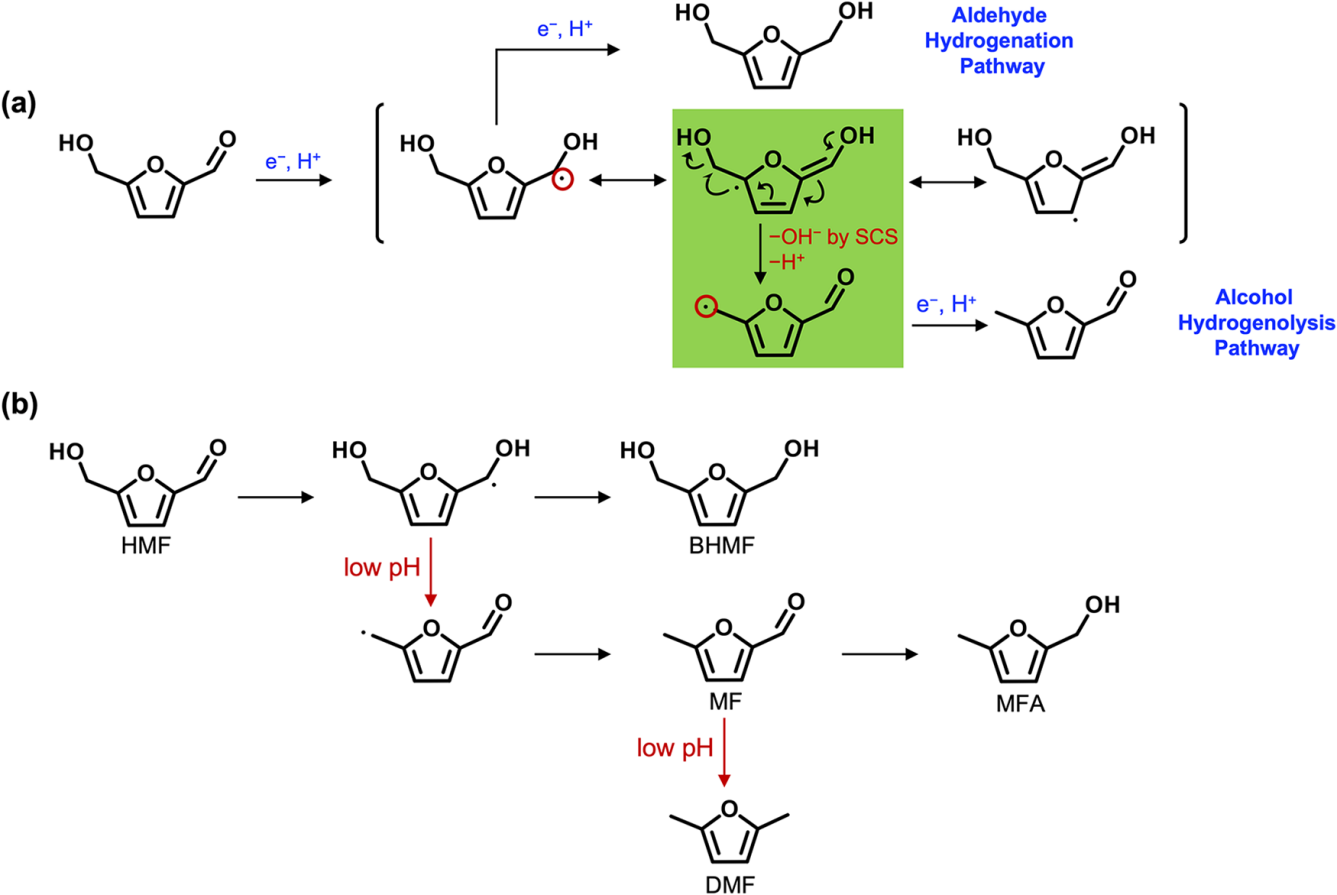
(a) Proposed model showing how alcohol hydrogenolysis of HMF occurs through ketyl radical formation, followed by SCS (green box). (b) Reduction of HMF to various products with steps facilitated in an acidic solution, indicated by red arrows. The electrode surface that stabilizes the reactants and intermediates is not shown for simplicity.

In contrast, the strong σ­(C–O_alcohol_) bond in BHMF has no mechanism for preactivation, and the direct cleavage of this bond involves an electron injection into its σ* orbital. However, the σ* orbital of the C–O_alcohol_ bond is generally much higher in energy than the π* orbital of the C=O bond. As a result, BHMF does not exhibit reactivity for alcohol hydrogenolysis within the potential range where a carbonyl group can be reduced. This explanation also applies to all other alcohol molecules discussed above that lack a carbonyl group.

We can also explain the lack of alcohol hydrogenolysis activity in molecules where the carbonyl group is replaced with −CF_3_, −COOR, or −COOH. One of our initial hypotheses was that the C–O_alcohol_ bond is the redox center for alcohol hydrogenolysis and this bond is activated by the electron-withdrawing nature of the carbonyl group. If that were the case, more strongly electron-withdrawing groups such as −CF_3_, −COOR, or −COOH should activate the C–O_alcohol_ bond to a greater extent. However, we now understand that alcohol hydrogenolysis proceeds through the reduction of the carbonyl group, and it is the SOMO of the resulting ketyl radical that activates the C–O_alcohol_ bond. Therefore, the critical factor is not the electron-withdrawing ability of the substituent, but rather its ability to be readily reduced to form a ketyl radical.

The −CF_3_ group lacks a carbonyl functionality, and the carbonyl groups in −COOR and −COOH groups are significantly more difficult to reduce than those in aldehydes and ketones. This is because the −OR and −OH groups directly attached to C_carbonyl_ can participate in resonance with the carbonyl group, stabilizing it and making its reduction to the ketyl radical more difficult (Figure S12).^44^ As a result, these groups remain inactive under the reduction conditions used in this study and cannot facilitate alcohol hydrogenolysis.

Finally, we note that it has been experimentally observed that the conversion of HMF to DMF, which requires hydrogenolysis of both the alcohol and aldehyde groups, is promoted in acidic solutions.^25,27,43^ While the effect of low pH on facilitating aldehyde hydrogenolysis over aldehyde hydrogenation during HMF reduction (i.e., formation of DMF vs MFA from MF) has been well explained in a previous study,^25^ no study had previously explained why low pH is necessary for alcohol hydrogenolysis of HMF prior to this work. This study successfully elucidates the effect of pH on the selectivity between alcohol hydrogenolysis and aldehyde hydrogenation of HMF (i.e., formation of MF vs BHMF) (Figure 9b), thereby completing our understanding of why the conversion of HMF to DMF with high selectivity requires acidic media.

## Conclusions

In summary, we designed and conducted systematic experiments to elucidate why hydroxyl groups in certain molecules, such as HMF, readily undergo alcohol hydrogenolysis, whereas those in other molecules, such as BHMF and MFA, remain inactive. We discovered that molecules susceptible to alcohol hydrogenolysis commonly contain a carbonyl group, with the carbonyl and hydroxyl groups arranged in a spatial configuration equivalent to that in an α-ketol, such as 2HAP. In an α-ketol, the ketyl radical formed during the first step of carbonyl reduction serves as a common intermediate for the formation of both carbonyl hydrogenation and alcohol hydrogenolysis products. The latter reaction occurs because the SOMO of the ketyl radical activates the C_α_–O_alcohol_ bond, leading to SCS. In HMF, the hydroxyl group is not directly attached to the C_α_ position of the carbonyl group; rather, it is attached to a carbon that is resonantly equivalent to the C_α_ position through the aromatic ring. As SCS occurs via extended π-conjugation, its role in enabling alcohol hydrogenolysis of HMF was difficult to recognize until the spatial and electronic relationship between the alcohol and aldehyde groups in HMF was revealed by this study.

We further showed that the feasibility of SCS involving the departure of the hydroxyl group from C_α_–or its equivalent–decreases with increasing pH. Therefore, pH is a critical factor affecting the selectivity between carbonyl hydrogenation and alcohol hydrogenolysis in aromatic α-ketols and their structural equivalents. The foundational understanding developed in this study coherently explains all experimental results presented here and in previous studies. Moreover, this study provides insights into recognizing previously hidden SCS processes in the electrochemical reduction of complex biomass-derived oxygenates and into the rational exploitation and control of this mechanism to tune reaction selectivity.

## Supplementary Material



## Notes

Scanning electron microscopy images of a Cu foam electrode before and after electrolysis; potential-dependent electrolysis results; HPLC and GC chromatograms; additional LSVs; further product quantification results; tables of FEs; resonance structures of carboxylic acids and esters; and computational results showing orbital overlaps (PDF)
