# Supplementary material for: Understanding Electrochemical Alcohol Hydrogenolysis Enabled by Carbonyl Reduction in Lignocellulosic Biomass-Derived Aromatic Oxygenates
Source: J Am Chem Soc. 2026 Jul 16;148(29):31393–404. doi: 10.1021/jacs.6c09300 (PMC13426308; doi:10.1021/jacs.6c09300)
Supplement: Supplementary file 1 [file ja6c09300_si_001.pdf]

Supporting Information

Understanding Electrochemical Alcohol Hydrogenolysis Enabled by Carbonyl Reduction in Lignocellulosic Biomass-Derived Aromatic Oxygenates

Myohwa Ko,+[a] Xin Yuan,+[a] Kwanpyung Lee,[a] J. R. Schmidt,*[a] and Kyoung-Shin Choi*[a]

[+] These authors contributed equally to this work.

[a] *Department of Chemistry, University of Wisconsin-Madison, Madison, WI 53706, USA*

[*] Email: schmidt@chem.wisc.edu, kschoi@chem.wisc.edu

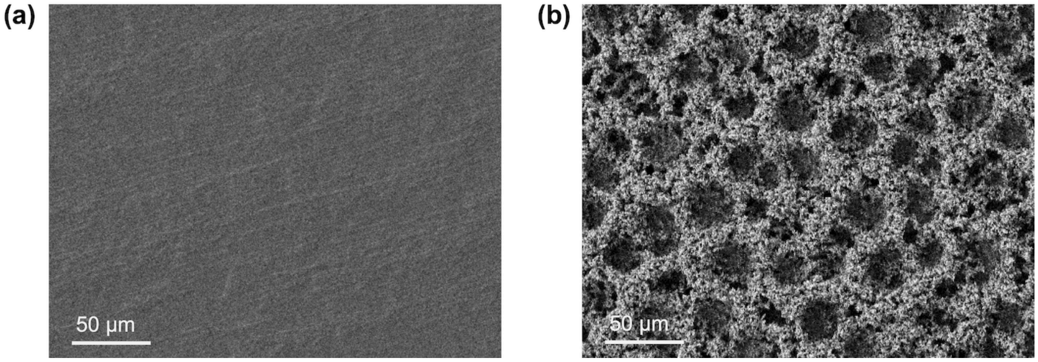

Figure S1. Scanning electron microscopy images of (a) bare Cu foil used as the substrate for electrodeposition of the Cu foam electrode and (b) the Cu foam electrode used as the working electrode for all LSV measurements and constant-potential reduction reactions in this study.

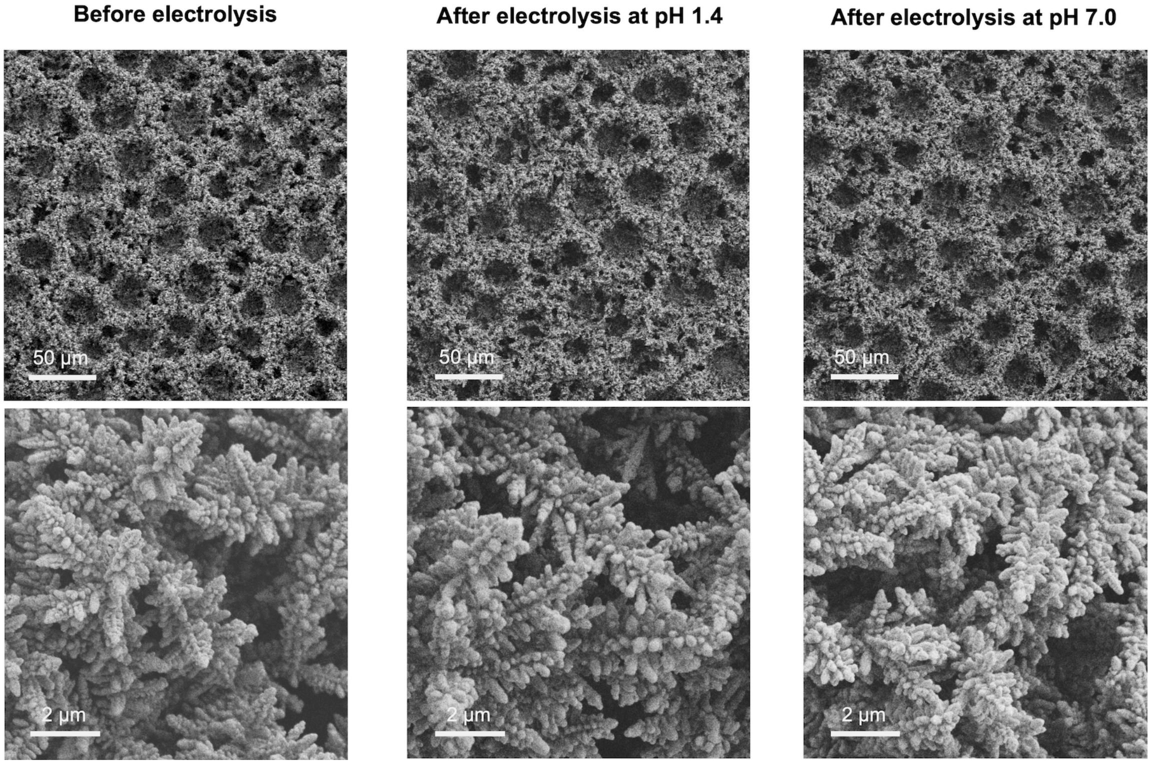

Figure S2. Low (top) and high (bottom) magnification scanning electron microscopy images of the Cu foam electrode before and after use in 2HAP reduction at –0.3 V vs. RHE in the solutions described in the main text.

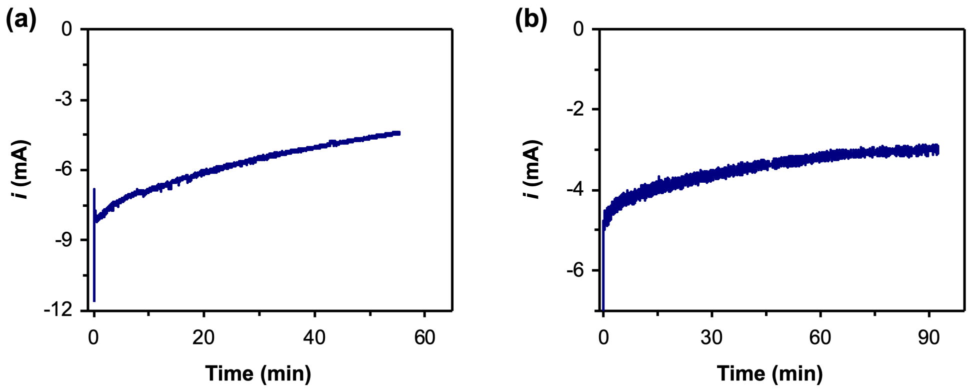

Figure S3. Current–time (*i*-*t*) plots during the reduction of 2HAP at –0.3 V vs. RHE (a) at pH 1.4 and (b) at pH 7 using Cu foam electrodes. The post-electrolysis SEM images of the Cu foam electrodes shown in Figure S2 were obtained after generating these *i*-*t* plots.

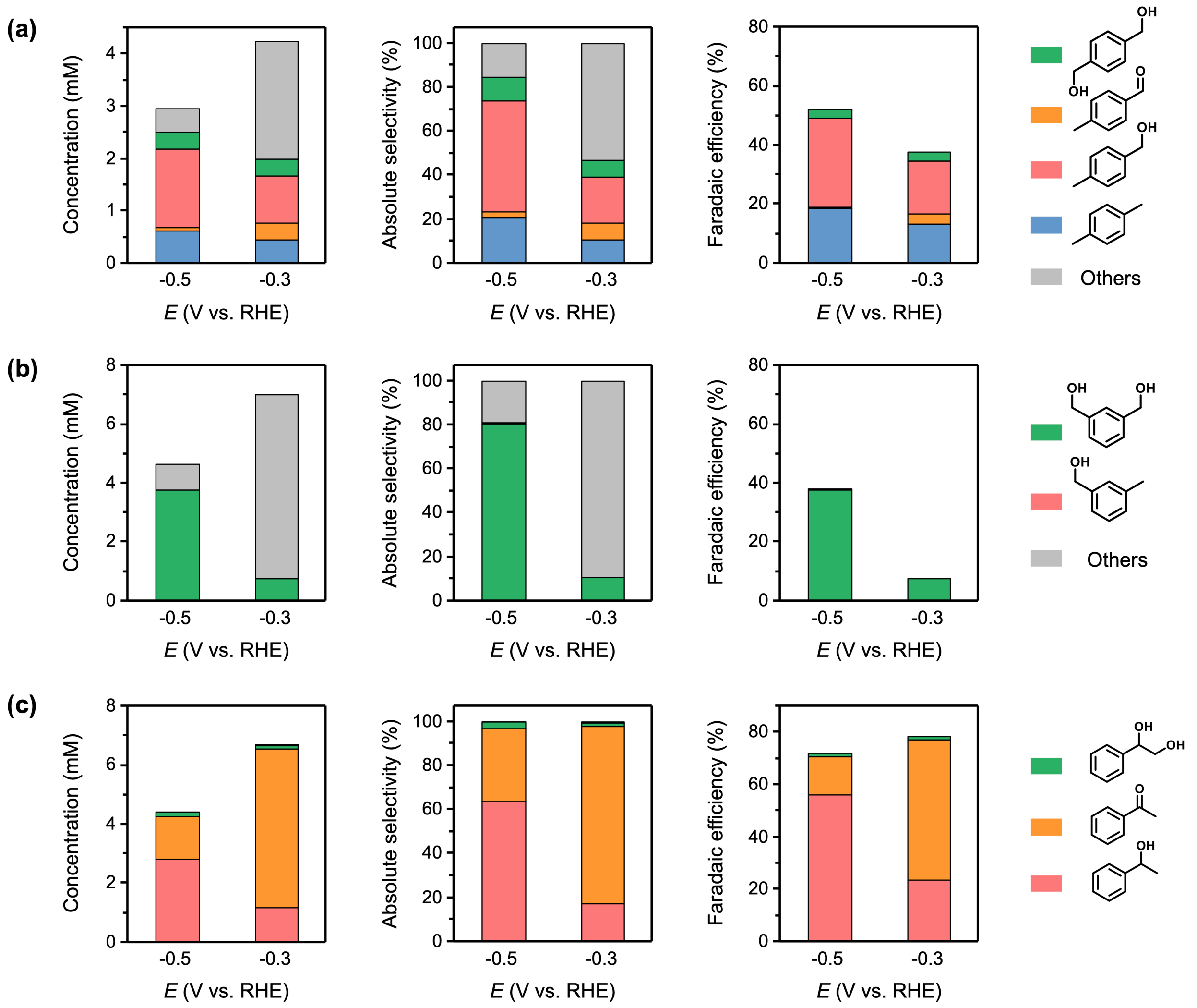

Figure S4. Product concentration, absolute selectivity (%), and FE (%) of (a) 4HMBAL, (b) 3HMBAL, and (c) 2HAP at –0.5 V and –0.3 V vs. RHE in the solutions described in the main text. For the case of 4HMBAL and 3HMBAL, the majority of the reactant at –0.3 V vs. RHE was consumed for radical dimerization (e.g., ketyl radical dimerization resulting in pinacol production), denoted as "others", which is why we chose to present the results obtained at –0.5 V vs. RHE in the main text. For the case of 2HAP, no detectable dimerization product was observed at either potential. Thus, we chose to present the results obtained at –0.3 V vs. RHE with less HER contribution in the main text.

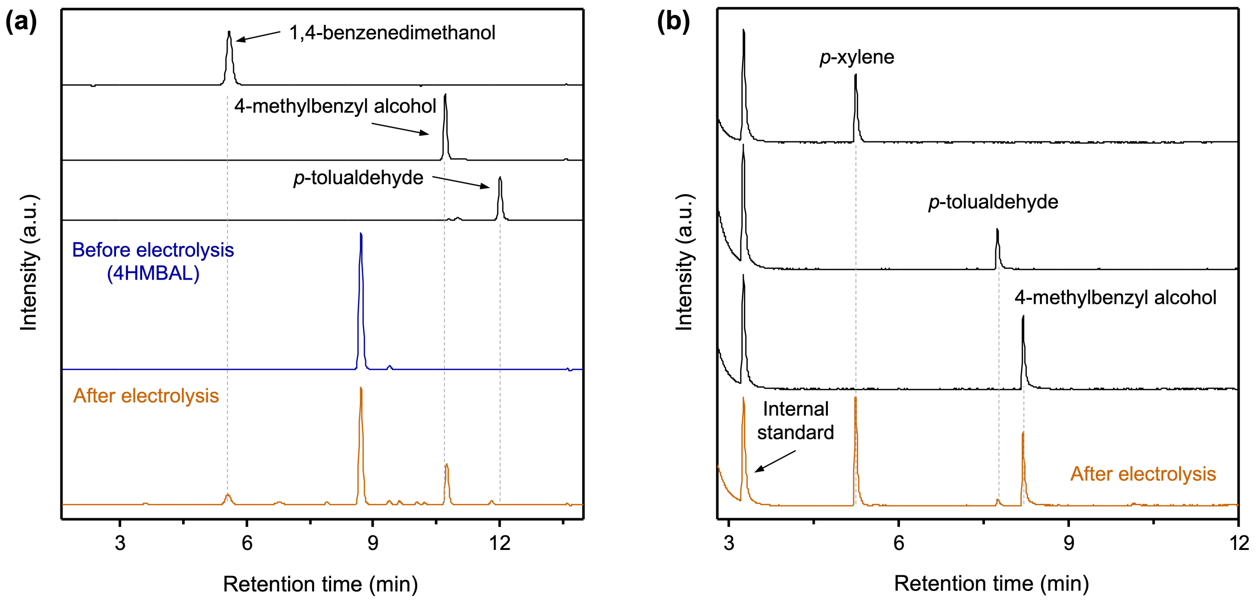

Figure S5. (a) HPLC chromatograms of the electrolyte before and after electrolysis of 10 mM 4HMBAL at –0.5 V vs. RHE. (b) GC chromatograms of the cyclohexane layer after electrolysis of 10 mM 4HMBAL at –0.5 V vs. RHE. Chromatograms of feasible, commercially available products are also shown. Unassigned peaks in (a) are most likely due to radical dimerization products, which are not commercially available.

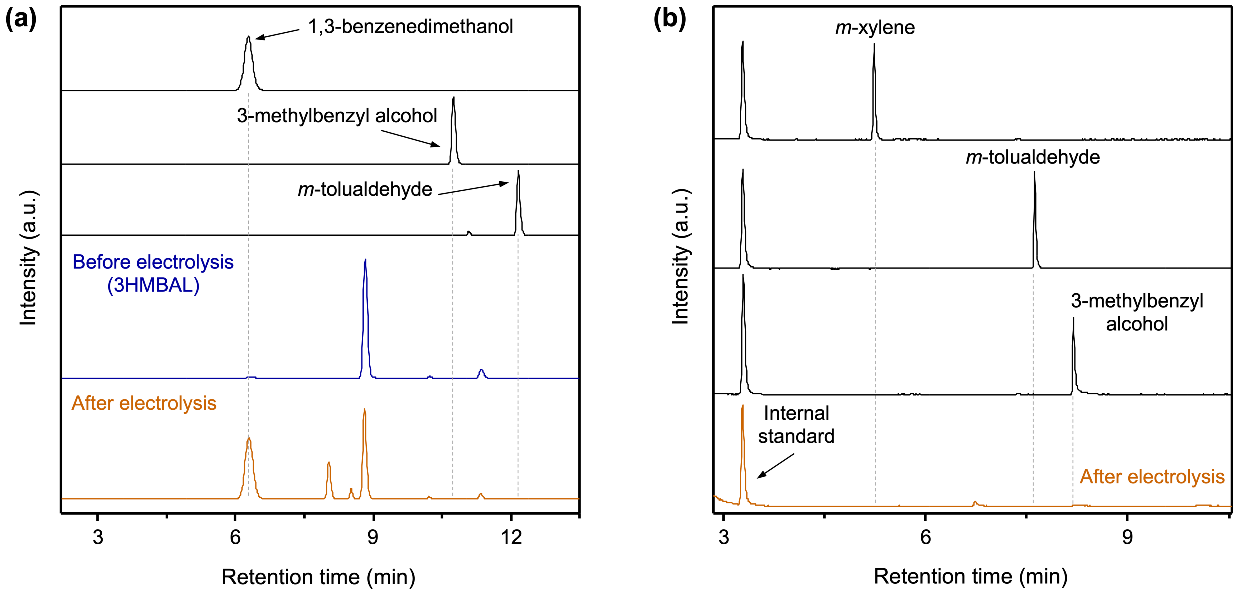

Figure S6. (a) HPLC chromatograms of the electrolyte before and after electrolysis of 10 mM 3HMBAL at –0.5 V vs. RHE. (b) GC chromatograms of the cyclohexane layer after electrolysis of 10 mM 3HMBAL at –0.5 V vs. RHE. Chromatograms of feasible, commercially available products are also shown.

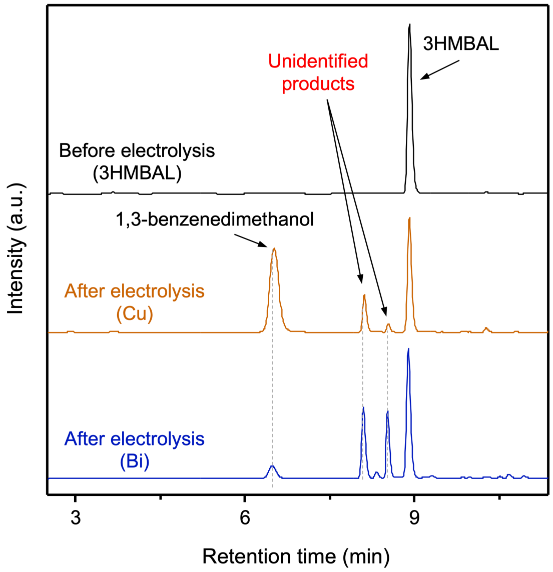

Figure S7. HPLC chromatograms of the electrolyte before and after electrolysis of 10 mM 3HMBAL using Cu and Bi electrodes. Bi has been reported to promote ketyl radical dimerization, thereby maximizing pinacol formation.[S1] The two previously unassigned peaks in Figure S6a are significantly enhanced when Bi is used instead of Cu, suggesting that they originate from the corresponding pinacol products, which are present as two diastereomers. The radical dimerization products of 3HMBAL and other reactants are not commercially available; therefore, this indirect comparison was used to infer their formation.

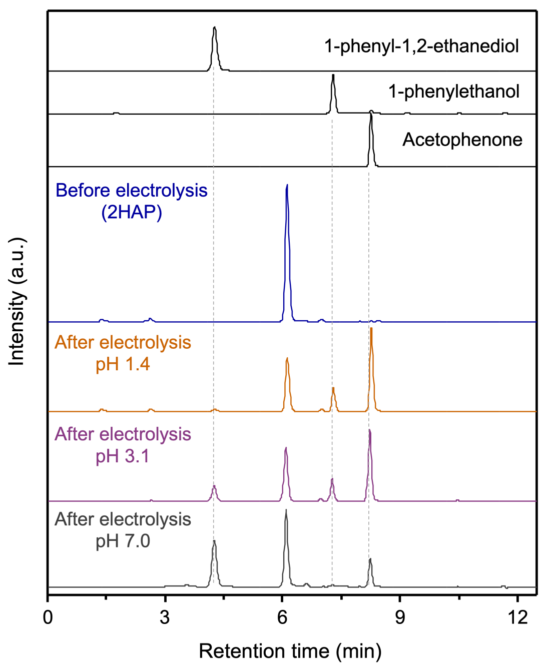

Figure S8. HPLC chromatograms of the electrolyte before and after electrolysis of 10 mM 2HAP at –0.3 V vs. RHE in pH 1.4, 3.1, and 7.0 buffer solutions. Chromatograms of feasible, commercially available products are also shown.

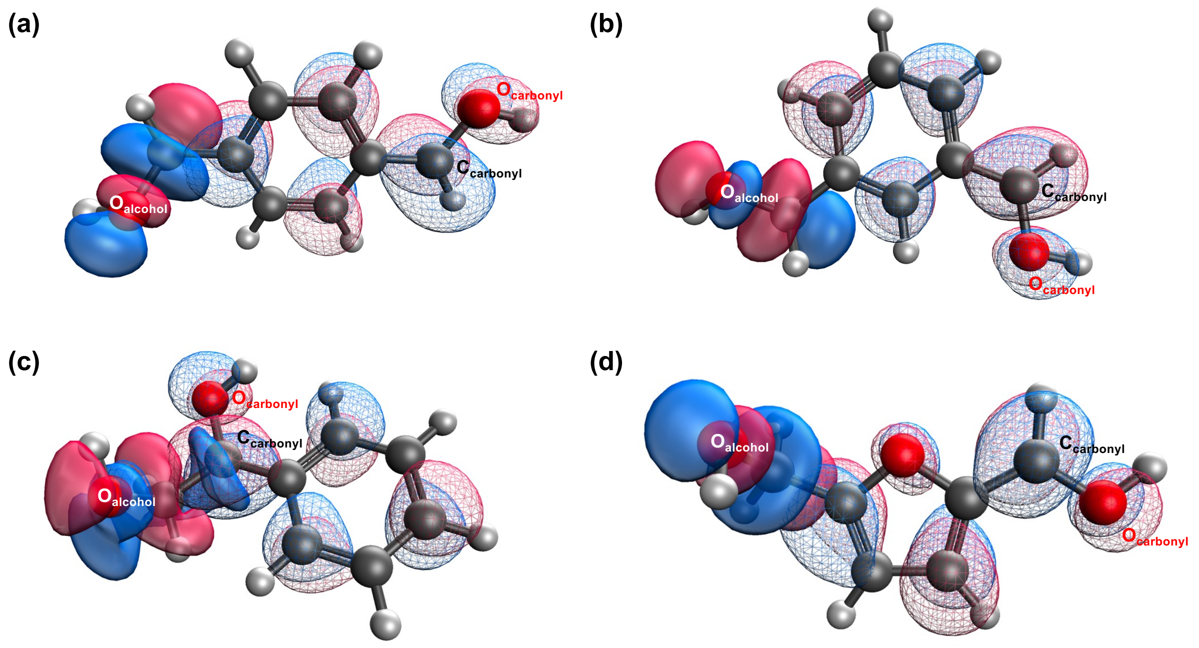

Figure S9. Isosurface plots of the σ*(C–$O_{alcohol}$) orbital (solid) and the SOMO (mesh) of the ketyl radical of (a) 4HMBAL, (b) 3HMBAL, (c) 2HAP, and (d) HMF. An isosurface level of 0.1 $Å^{-3}$ is used (dark gray: carbon, light gray: hydrogen, red: oxygen). Overlap between the σ*(C–$O_{alcohol}$) orbital and the SOMO is observed for (a), (c), and (d), but not for (b).

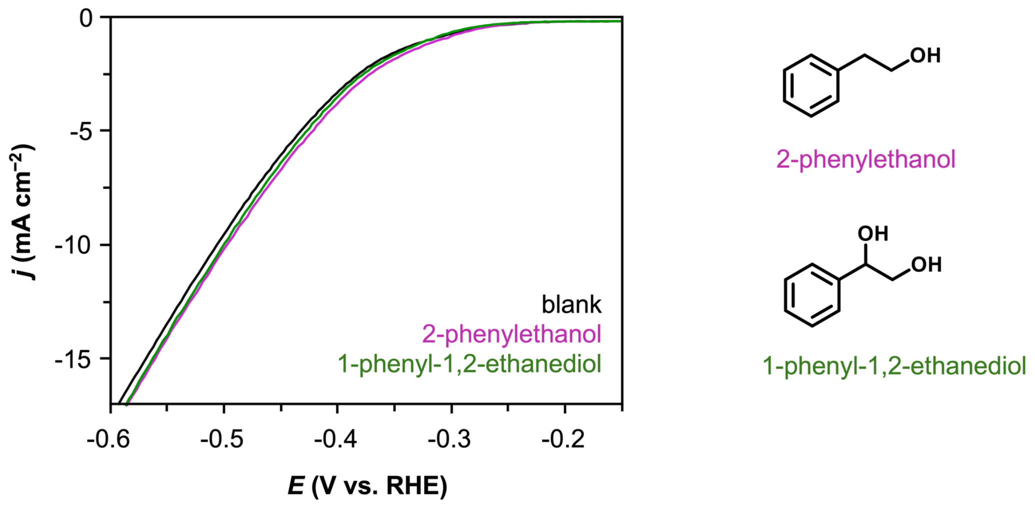

Figure S10. LSVs recorded in the absence of reactant (blank) and with 10 mM 2-phenylethanol or 1-phenyl-1,2-ethanediol using a Cu foam electrode in a pH 1.4 buffered solution.

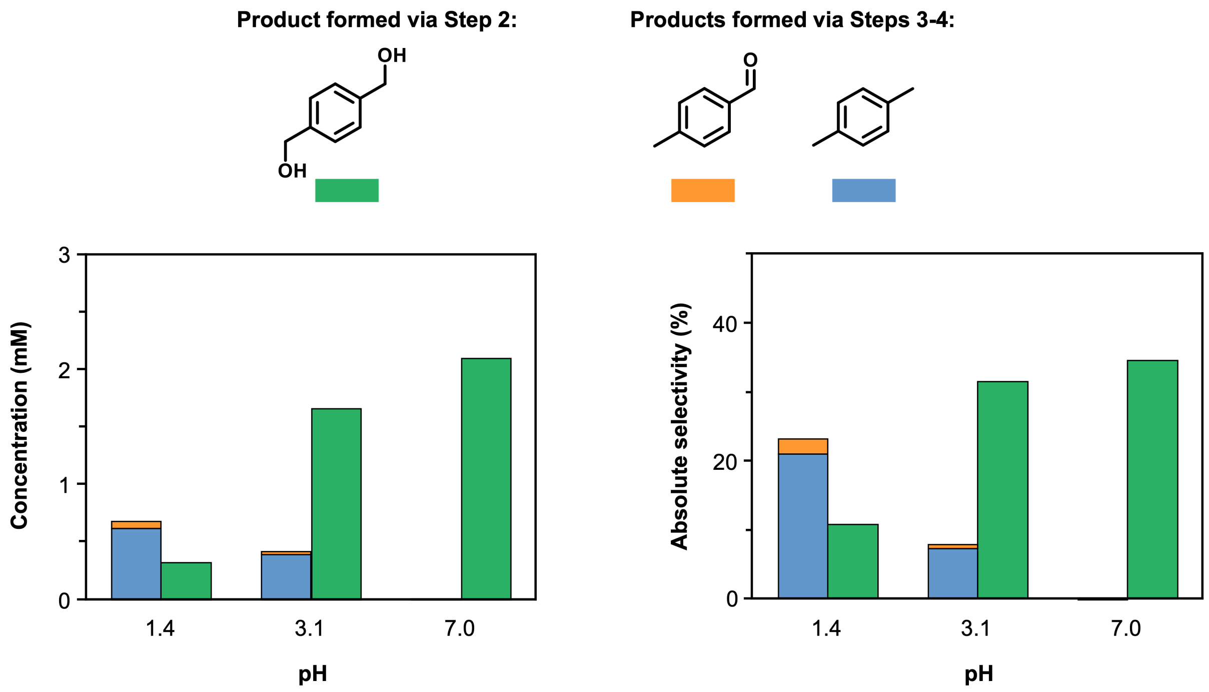

Figure S11. Quantification of products obtained from constant potential electrolysis of 10 mM 4HMBAL at –0.5 V vs. RHE after passing the amount of charge equivalent to $2e^-$ per 4HMBAL molecule using a Cu foam electrode in pH 1.4, 3.1, and 7.0 buffer solutions. Steps 2, 3 and 4 in the legend are those from Figure 8a in the main text. Note that 4-methylbenzyl alcohol was also produced; however, this product was not included here because it can form with or without alcohol hydrogenolysis.

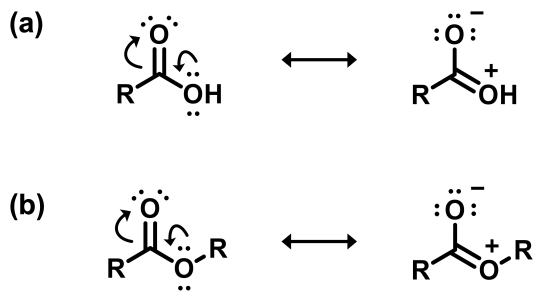

Figure S12. Resonance structures of the carbonyl groups in carboxylic acids and esters enabled by the –OH and –OR groups directly attached to $C_{carbonyl}$, which stabilize the carbonyl groups, making their reduction to the ketyl radical difficult.

Table S1. Faradaic efficiency of products obtained from constant-potential electrolysis of 10 mM 4HMBAL in a pH 1.4 buffer solution at –0.5 V vs. RHE using a Cu foam electrode after passing the amount of charge equivalent to $2e^-$ per 4HMBAL molecule.

Reactant	Products	FE (%)
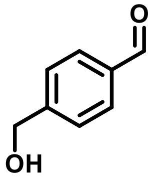	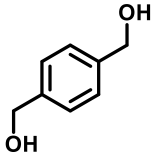	3.2
	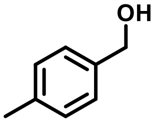	29.9
	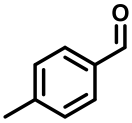	0.6
	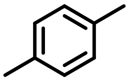	18.6

Table S2. Faradaic efficiency of products obtained from constant-potential electrolysis of 10 mM 3HMBAL in a pH 1.4 buffer solution at –0.5 V vs. RHE using a Cu foam electrode after passing the amount of charge equivalent to $2e^-$ per 3HMBAL molecule.

Reactant	Products	FE (%)
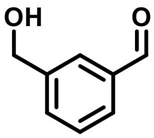	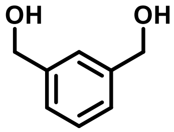	37.5
	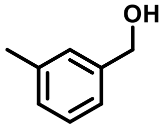	0.5
	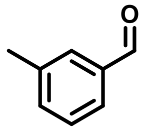	0 (BDL[a])
	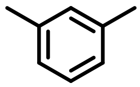	0 (BDL)

[a] BDL: below detection limit

Table S3. Faradaic efficiency of products obtained from constant-potential electrolysis of 10 mM 2HAP at –0.3 V vs. RHE after passing the amount of charge equivalent to 2e⁻ per 2HAP molecule using a Cu foam electrode in pH 1.4, 3.1, and 7.0 buffer solutions.

pH /Reactant	Products	FE (%)
1.4 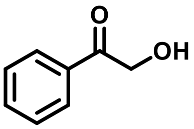	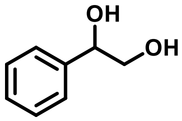	1.7 ± 0.4
	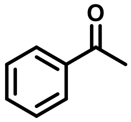	53.1 ± 2.3
	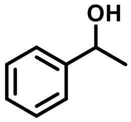	21.2 ± 2.5
3.1 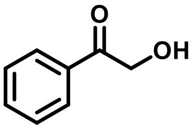	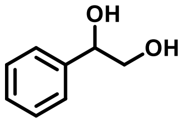	8.2 ± 0.3
	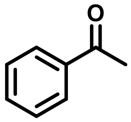	45.6 ± 1.9
	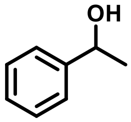	22.0 ± 4.4
7.0 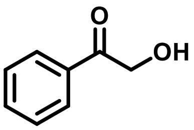	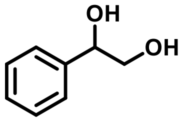	32.1 ± 2.1
	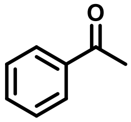	20.1 ± 5.0
	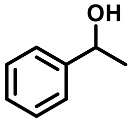	1.3 ± 0.1

Table S4. Quantification of products obtained from constant-potential electrolysis of 10 mM 4-methylbenzyl alcohol or 1,4-benzenedimethanol in a pH 1.4 buffer solution at –0.5 V vs. RHE using a Cu foam electrode after passing the amount of charge equivalent to $2e^-$ per reactant.

Reactants[a]	Products	Yield (mM)
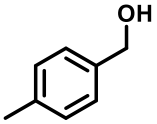 4-methylbenzyl alcohol	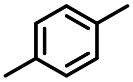	0 (BDL[b])
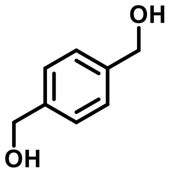 1,4-benzenedimethanol	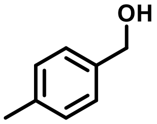	0 (BDL)
	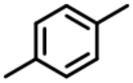	0 (BDL)

[a] The conversion of the reactant was below 1%.
[b] BDL: below detection limit

Table S5. Quantification of products obtained from constant-potential electrolysis of 10 mM (4-(trifluoromethyl)phenyl)methanol, 4-(hydroxymethyl)benzoic acid, or methyl 4-(hydroxymethyl)benzoate in a pH 1.4 buffer solution at –0.5 V vs. RHE using a Cu foam electrode after passing the amount of charge equivalent to $2e^-$ per reactant.

Reactants[a]	Products	Yield (mM)
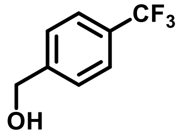 (4-(trifluoromethyl)phenyl)methanol	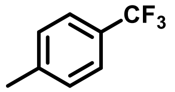	0 (BDL[b])
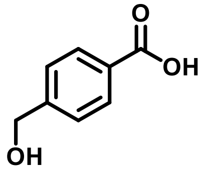 4-(hydroxymethyl)benzoic acid	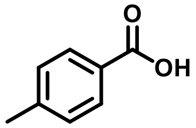	0 (BDL)
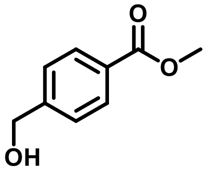 methyl 4-(hydroxymethyl)benzoate	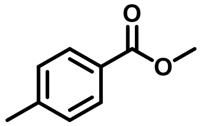	0 (BDL)

[a] The conversion of the reactant was below 1%.
[b] BDL: below detection limit

Table S6. Quantification of products obtained from constant-potential electrolysis of 10 mM 2-phenylethanol or 1-phenyl-1,2-ethanediol in a pH 1.4 buffer solution at –0.5 V vs. RHE using a Cu foam electrode after passing the amount of charge equivalent to 2e⁻ per reactant.

Reactants[a]	Products	Yield (mM)
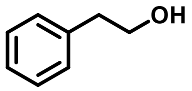 2-phenylethanol	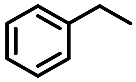	0 (BDL[b])
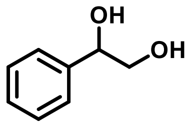 1-phenyl-1,2-ethanediol	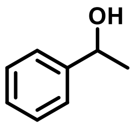	0 (BDL)
	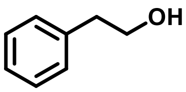	0 (BDL)
	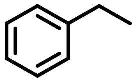	0 (BDL)

[a] The conversion of the reactant was below 1%.
[b] BDL: below detection limit

References

S1. Eisenberg, J. B.; Lee, K.; Schmidt, J. R.; Choi, K.-S., Understanding the Competition between Alcohol Formation and Dimerization during Electrochemical Reduction of Aromatic Carbonyl Compounds. *J. Am. Chem. Soc.* **2025,** *147* (45), 41390-41403.
